# LAPAROSCOPIC ANTIREFLUX SURGERY: ARE OLD QUESTIONS ANSWERED? MESH HERNIOPLASTY

**DOI:** 10.1590/0102-672020220002e1710

**Published:** 2023-01-09

**Authors:** Adham Raja SAAD, Vic VELANOVICH

**Affiliations:** 1 University of South Florida, Division of Gastrointestinal Surgery, Morsani College of Medicine – Tampa, Florida, USA.

**Keywords:** Gastroesophageal Reflux, Hernia, Hiatal, Laparoscopy, Recurrence, Surgical Mesh, Refluxo Gastroesofágico, Hérnia Hiatal, Laparoscopia, Recidiva, Telas Cirúrgicas

## Abstract

Hiatal hernias are at high risk of recurrence. Mesh reinforcement after primary approximation of the hiatal crura has been advocated to reduce this risk of recurrence, analogous to mesh repair of abdominal wall hernias. However, the results of such repairs have been mixed, at best. In addition, repairs using some type of mesh have led to significant complications, such as erosion and esophageal stricture. At present, there is no consensus as to (1) whether mesh should be used, (2) indications for use, (3) the type of mesh, and (4) in what configuration. This lack of consensus is likely secondary to the notion that recurrence occurs at the site of crural approximation. We have explored the theory that many, if not most, “recurrences” occur in the anterior and left lateral aspects of the hiatus, normally where the mesh is not placed. We theorized that “recurrence” actually represents progression of the hernia, rather than a true recurrence. This has led to our development of a new mesh configuration to enhance the tensile strength of the hiatus and counteract continued stresses from intra-abdominal pressure.

## RESUMO

As hérnias hiatais têm alto risco de recidiva. O reforço com tela após a aproximação primária dos pilares hiatais tem sido defendido, para reduzir esse risco de recidiva, análogo ao reparo com tela de hérnias da parede abdominal. No entanto, os resultados de tais reparos foram variados, na melhor das hipóteses. Além disso, os reparos com algum tipo de tela levaram a complicações importantes, como erosão e estenose esofágica. Atualmente, não há consenso sobre 1) se a tela deve ser usada, 2) indicações para uso, 3) o tipo de tela e 4) em qual configuração. Essa falta de consenso provavelmente é secundária à noção de que a recidiva ocorre no local da aproximação crural. Exploramos a teoria de que muitas, se não a maioria, das recidivas ocorrem nas faces anterior e lateral esquerda do hiato, normalmente onde a tela não é colocada. Nós teorizamos que a “recidiva” na verdade representa a progressão da hérnia, em vez de uma verdadeira recidiva, levando ao desenvolvimento de uma nova configuração da tela, para aumentar a resistência à tração do hiato e neutralizar as tensões contínuas da pressão intra-abdominal.

## INTRODUCTION

The customary repair of hiatal hernia is composed of reduction of the stomach from the posterior mediastinum, excision of the hernia sac, and approximation of the hiatal crura around the esophagus, with or without a fundoplication^23,24^. Unfortunately, these hernias are prone to recurrence after repair^1,3,12^. Studies found that repair of recurrent hiatal hernia is becoming a more frequent indication for hiatal hernia surgery^3,24,25^.

With the acceptance of mesh as a routine adjunct to abdominal wall hernia repair, it would seem a natural extension that mesh would lead to a reduction in hiatal hernia recurrence. Kuster and Gilroy^10^ were the first to study with mesh paraesophageal hernia repair in 1993. Subsequently, mesh repair has gained in popularity, although not universally accepted as necessary for hiatal hernia repairs^20^. In fact, not only is there no consensus on whether to use mesh, but there is also no agreement on the type of mesh material to use or its configuration^6,20^.

Our purpose in this review was to assess the present status of mesh used in hiatal hernioplasty, a new theory of why these hernias recur, and future directions for mesh use.

### Rationale and Present Uses of Mesh

The underlying rationale for the use of mesh in hiatal hernioplasty originated as a concept transfer from the tension-free repair of abdominal wall hernias. The concept is that mesh placement at the hiatus removed or decreased tension at the site of crural approximation. That is, there is increased stress on the tissues due to lateral strain on the right and left crura at the site of the cruroplasty sutures. In fact, the tension on this suture approximation has been measured with tensometers to assess the efficacy of reducing the strain of both relaxing incisions^2^ and biological mesh repair^21^. Therefore, it has been theorized that suture approximation should be reinforced with mesh to combat this relentless tension.

**Figure 1.**
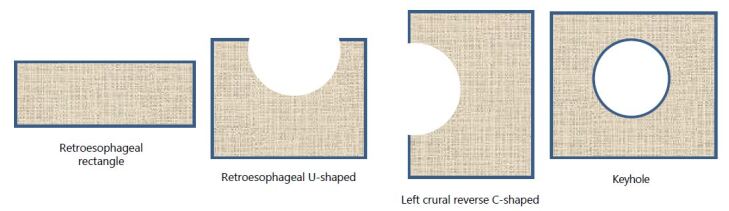
Basic mesh configurations used in mesh hiatal hernia repair.

As mentioned previously, there is no consensus as to the type of mesh material or its configuration. Our purpose here is not to enumerate the different mesh products available and used at the hiatus; however, they can be broadly characterized as permanent materials that will never reabsorb and resorbable materials that provide scaffolding for tissue ingrowth^7^. As far as configuration, there are generally four basic types (Figure 1). The retroesophageal bar-/rectangle-shaped mesh is placed to cover the primary suture from the right to the left crura. The retroesophageal U-shaped mesh is placed similarly to the bar-shaped one but extends up to the right and left crural pillars. The reverse C-shaped mesh is placed with the lower horizontal portion covering the primary suture repair from the right to the left crus, the vertical portion extending up the left crus, and the upper horizontal portion extending anterior to the esophagus from the left to the right crus. Finally, the keyhole-shaped mesh completely encircles the esophagus. A basic principle is that all are onlay mesh placements over a primary suture repair. Although there is some information on “bridging” mesh placements from the left to the right crura, this type of repair has not gained wide popularity^9,13,18,19^.

### Present Status of Mesh Data

It is beyond the scope of this article to review all the available data on mesh hiatal hernioplasty, but an analysis of the available meta-analyses/systematic reviews is profitable. A PubMed search of the published systematic reviews or meta-analyses yielded 22 articles published from 2011 to 2021. Of these, five were published in 2020 or 2021. Campos et al.^4^ reviewed eight articles for the systematic review and seven for the meta-analysis comparing mesh hernioplasty to primary suture repair. They found no statistically significant differences in recurrence rate, postoperative complications, intraoperative complications, deaths, or reoperations. Rausa et al.^17^ reviewed 17 articles studying 1,857 patients comparing absorbable to nonabsorbable mesh. They found that the relative risk of recurrence was higher for absorbable mesh (odds ratio 2.3 [95% confidence interval 0.8–6.3]) and primary repair (odds ratio 3.6 [95% confidence interval 2.0–8.3]) compared to nonabsorbable mesh. Petric et al.^16^ reviewed seven randomized controlled trials of mesh versus suture repair containing 735 patients. They found no statistically significant differences in short-term follow-up (6–12 months), 10.1% recurrence rate for mesh versus 15.5% for primary repair, or long-term follow-up (3–5 years), 30.7% mesh versus 31.3% primary. Laxague et al.^11^ in their review of 53 studies from 2000 to 2020 concluded that the available data are quite heterogeneous and generally failed to demonstrate definitively the superiority of either mesh or suture repair. Finally, Spiro et al.^22^ in a systematic review of mesh complications including 35 case reports/series of 74 patients and 20 observational studies of 75 complications in over 4,200 patients repaired with mesh found an erosion rate of 0.035%, with polytetrafluoroethylene being the most reported. Our group previously published a decision analysis of mesh versus primary suture repair of paraesophageal hernia using a utility-based scoring system and did not find that one repair was significantly better than the other^13^. These new data do not appear to change that conclusion.

### Theory of Hiatal Hernia Recurrence

The basic question is: Why do hiatal hernias recur? The answer relates to both their origin and the manner in which they were repaired.

**Figure 2.**
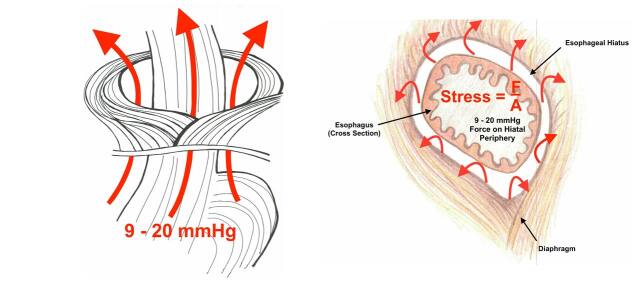
Transhiatal pressures acting upon the hiatal crura leading to strain upon the hiatus. From Saad and Velanovich^19^.

We have previously published our theory on hiatal hernia recurrence^23^ based on our observations on the shape and location of these recurrences^21^. Although it is not our intention here to recapitulate the entire theory, the summary of it is that the innate tensile strength of the hiatal tissue is overcome by the unrelenting pressure differential between the intra-abdominal compartment and the intra-thoracic compartment (Figure 2). This theory is similar to the finding of Del Grande et al.^5^ with respect to the pathophysiology of gastroesophageal reflux.

Repair of a hiatal hernia is relatively unique in the spectrum of hernia repairs. Unlike abdominal wall hernias where the defect is completely closed, with hiatal hernias, even after repair, an opening must remain to allow for the passage of the esophagus from the chest into the abdomen. In this way, it is similar to parastomal hernia repairs, which are also notorious for recurrence^8^. Due to this opening, the forces that caused the enlargement of the hiatal orifice initially are still acting on it even after repair. This pressure leads to strain on the hiatal tissue. When the strain overcomes the yield strength of the tissue, there is a permanent deformity in the hiatal crura, leading to the hernia. The yield strength of the hiatal crura is related to the biomechanical properties of the tissue. If the yield strength is low, it will not take much stress to cause the deformity. If the yield strength is high, much higher stresses are required. Therefore, reducing hiatal hernia recurrence can be accomplished by either reducing the pressures acting upon the hiatus (e.g., weight loss in the obese) or increasing the yield strength of the tissue (e.g., mesh reinforcement).

In order for mesh reinforcement to adequately increase yield strength, it must be placed where the forces are acting upon the tissue. We^9,24^ as well as others^14,15^have shown that the U-shaped configuration appears to be inadequate, with recurrence rates similar to those of primary repair. Interestingly, as an aside, when mesh has been placed, hernias do not appear to be as symptomatic when they recur^23,24^. We concluded that the U-shaped and bar-shaped configurations are inadequate as reinforcement because they do not reinforce the area where most recurrences occur, anterior and to the left. This led us to change our mesh configuration from the U-shaped to the keyhole pattern. With this pattern, we have noted a lower recurrence rate^9^. Nevertheless, we still believed there was space for improvement.

**Figure 3.**
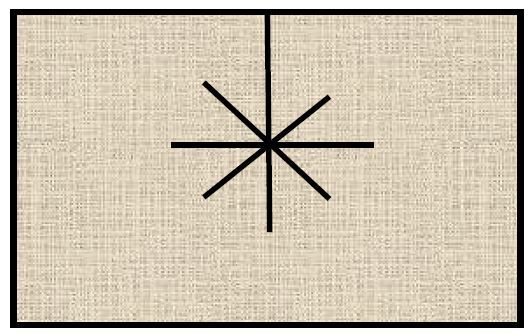
The “starburst” mesh pattern.

**Figure 4.**
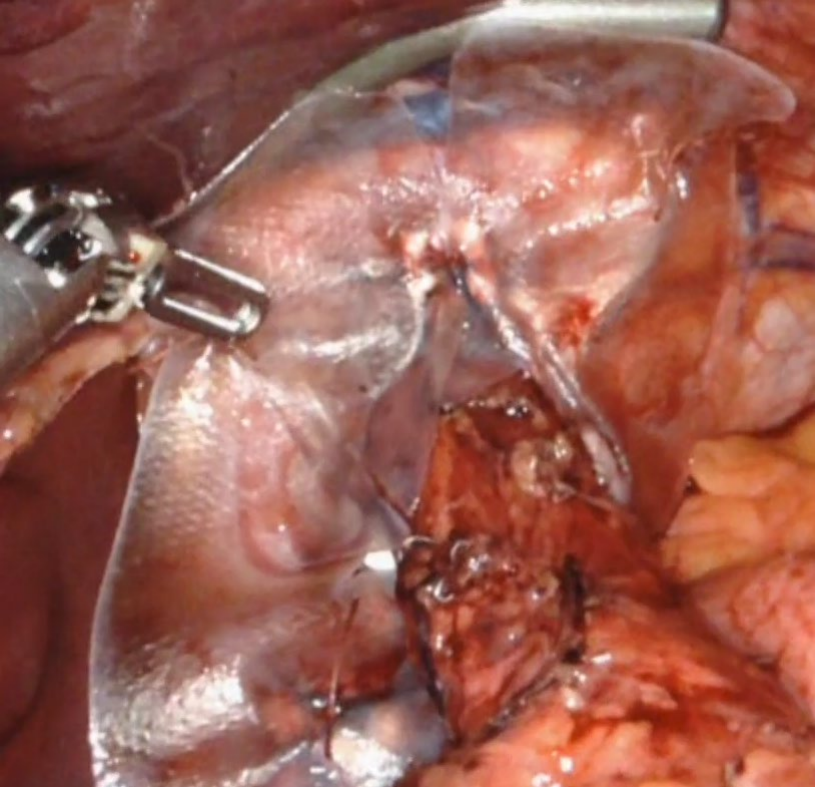
Intraoperative photograph of starburst mesh placement.

This led us to develop the “starburst pattern” of mesh configuration^20^. The concept is to increase the tensile strength of the tissue of the hiatal opening. With this, a biological mesh is cut so that 8 pie-shaped phalanges of mesh are created (Figure 3). The mesh is placed around the esophagus after primary suture repair of the hiatal defect in a keyhole fashion with the phalanges folded over the edges of the hiatal opening (Figure 4). Our preliminary results are encouraging^20,21^.

### Future Directions

Prevention of the recurrence of hiatal hernias is a function of increasing the tensile strength of the hiatal tissue and/or reducing the transdiaphragmatic pressure on the tissue. Of course, it goes without saying that good surgical technique in hiatal hernia repair is mandatory. We have evolved to a practice of using mesh only in patients at “high risk” of recurrence, acknowledging that these risk factors are not universally accepted. Although our preference is the use of a biological material, we acknowledge that there is no consensus on material choice and further studies are needed to determine the optimal material. Our present practice is that when we use mesh, we use a keyhole configuration with the starburst pattern. Obviously, further studies are needed to determine if this, indeed, is the optimal configuration. Until adequately powered trials are conducted, controversy and debate on the use of mesh in hiatal hernia repair will continue.
